# History and Pathology of Vaccination

**Published:** 1890-03-29

**Authors:** 


### THE PRACTITIONER'S BOOKSHELF.

(Books for Review should be sent to The Editor, The Lodge, Porchester Square, W.)

### HISTORY AND PATHOLOGY OF VACCINATION.*

It is a singular thing that a disbelief in the value of vaccination would appear to be accompanied in all cases, or in almost all, by a corresponding disbelief in the ability and the honesty of Jenner. From the anti-vaccinators who rave and rant in the columns of the *Vaccination Inquirer*, up to Dr. Creighton and Mr. Crookshank, all alike seem filled not only with opinions adverse to the prophylactic means, but with the utmost contempt, and even hatred of its great promulgator. Like the rest, Professor Crookshank has faith neither in Jenner nor vaccination, and he has spent much time, and taken a great deal of trouble, to show the former to be untrustworthy, and the latter worthless. To begin with, probably most people will attach more weight to his opinions on pathology than to his views of personal character, as the post he occupies at King's College gives him a certain right to be heard on the former subject, while we do not suppose that he claims his judgment to be better than that of anyone else on the latter question; nor do we think that a perusal of his history of Jenner will result in the opinion that he is likely to make any approach to an impartial historian.

In 1887, Professor Crookshank discovered an outbreak of Cow disease in a farm in Wiltshire, which he concluded to be the cow-pox. About the same time he had a discussion with Professor Klein, of the Local Government Board, as to cow scarlatina, and he appears to have alleged that what Klein thought scarlatina, was really cow-pox—it being understood, however, that the cases Klein saw did not belong to the Wiltshire outbreak. At the same time also Dr. Creighton had just published his work on "The Natural History of Cow-Pox and Vaccinal Syphilis." Thus was Professor Crookshank led into an investigation of the subject, and his main conclusion is, that the Government medical department is wrong, not only about scarlatina, but also about vaccination, and that Creighton is right in supposing that cow-pox has more to do with syphilis than with smallpox.

It is to be observed that various investigators have in past times, studied the relationship of cow-pox and smallpox, and have examined the disease as it is exhibited in casual outbreaks on bovine animals. This is notably the case with the late Mr. Ceely, of Aylesbury, whose writings are very largely quoted by both Creighton and Crookshank, and to the correctness of whose observations the utmost credence is given. It is also to be observed that neither Ceely, nor any of these earlier writers, seem to have been led by their inquiries unto the *fons et origo* of vaccination, to entertain the slightest doubt of its protective influence against smallpox, or the slightest suspicion that it had any relationship to venereal disease. Ceely indeed, whose classical papers on this subject are models of patient investigation and unbiassed observation, had the most absolute certainty of the antivariolous power of cow-pox, and the utmost admiration for the ability and acumen of Jenner in overcoming the practical difficulties of the subject—and this certainty and admiration he had at a time antecedent to that later inquiry of his, by which he established, as he believed, the doctrine that cow-pox was smallpox of the cow.

How then are we to account for this all-important practical difference between the older observers, and the two recent writers? We fear that an answer must be given unfavourable to the latter. The former were guided by facts, and the latter by theory. Ceely wrote only fifteen years after the death of Jenner, and the men of that generation were, many of them, old enough to recollect the revolution which vaccination had wrought, and had ample experience in their own dealings with the smallpox which yet remained, of the value of vaccination both in the comparative immunity from attack which it gave, and in the mildness of the disease where an attack did occur. These were days in which treatment in hospital was the exception, not the rule, and in which, therefore, the private practitioner had many opportunities of observing the doings of smallpox among both vaccinated and unvaccinated—opportunities which are now enjoyed only by the physicians of the great smallpox hospitals. The result was that almost every member of the profession then had the same well-founded reliance on good vaccination that every hospital physician has now. It is a most significant fact that we find no such theorists as Dr. Creighton and Mr. Crookshank among those who, in the present day, have had most to do with actual smallpox. They have seen and they know—while Professor Crookshank has to be content with such *a priori* reasoning as may result from his observations of cow disease in Wiltshire, and from readings on the subject of horse-pox, goat-pox, sheep-pox, and the like. We conceive it possible, however, that the theorists may in a roundabout way do something to help the next generation of Englishmen to obtain that practical knowledge of the

* "History and Pathology of Vaccination." By Edgar M. Crookshank, M.P. (London: H. K. Lewis, 136, Gower Street, W.C.)

benefits of vaccination which was possessed by people who lived half a century ago. For if the members of the Royal Commission now sitting, should be influenced by the prejudice against vaccination which they may find to exist, and which is bound to be fostered by the writings of Messrs. Creighton and Crookshank—if the commission, while itself having no doubt as to the value of vaccination, should yet believe it better to yield to the conscientious objections of its opponents by lessening the stringency of the law, then certainly we would see the beginning of a most tremendous experiment in which would be involved the health and life of tens of thousands of English children. As to the subjects of such an experiment this much is certain—that they would not be found in the families of medical men, for though compulsion were done away with to-morrow, they would continue to practise it with undiminished vigilance on their own children, and on all over whom the weight of their opinion might prevail.

The difference between Ceely and all other writers on the one hand, and Creighton and Crookshank on the other, is partly this. In regard to natural cow-pox, and to cow-pox on the hands of the dairymaids, the former held that a distinction was to be drawn between the symptoms of the specific infection, and the symptoms due to accidental causes—to dirt, to friction, to injury of the vesicles, and so on. The uncomplicated cow-pox he held to be a comparatively mild disease, while the ulcers, inflammation, &c., which often accompanied it on the persons of the milkers were due, he believed, to such other factors as have just been indicated. Creighton and Crookshank take the very reverse view. Creighton says that without severity there is no infective power, and that the ulcers, &c., were an essential part of the disease. Moreover, when ulcers occur in the present day they are not even now due to accident or carelessness, but are the result of what he calls unconscious memory—a return to the original severity of the disease as it occurred on the cow and on the milkers. This severity, too, is comparable to the severity of syphilis, and cow-pox is the malady in the cow corresponding to syphilis in man. Now, syphilis is no preventive of smallpox, and neither is cow-pox. To this view Professor Crookshank gives a general acquiescence. Taking a common-sense look at the matter, we can have little hesitation in concluding that Jenner and Ceely and the other observers before Creighton's time were right in holding that the essential symptoms of cow-pox were comparatively mild, and had nothing to do with the abscesses, erysipelas, &c., which might be brought on by the manifold filth and abuse to which the vesicles were subject on the cow's paps and the dairymaid's fingers. So, too, Creighton's theory seems far-fetched and altogether improbable. To accept it would involve us in quite a number of untenable opinions. We would need to hold that the wide spread traditions of the farmers were baseless; that the great host of tests performed by Jenner's contemporaries—tests of inoculating with smallpox those who had been vaccinated, and of exposing them to atmospheric infection—were fallacious; that the extraordinary protection afforded to smallpox nurses (which must be a matter of experience to some of our own readers) by re-vaccination is all a matter of chance; that the differences observable between the death-rate of the vaccinated and unvaccinated in smallpox hospitals have nothing to do with vaccination, and that, in short, the whole world for these hundred years back has been given up to the most astounding delusion that ever befooled the human brain; while we would also need to accept as true and as explanatory of all the various phenomena of vaccination, the fanciful doctrine that a specific cow disease can be engendered in a few days by a little carelessness and rough treatment on the part of the milker, and that the pathological process once engendered becomes endowed with powers of memory capable of carrying it back from the arm of a child in the year 1890 to the pap of a cow in the year 1798. To set aside all that we know of vaccination and smallpox in favour of this curious hypothesis would be to leave the safe paths of knowledge and experience, and to put ourselves under the guidance of a will-o'-the-wisp which would lead us only into bogs and pit-falls.

We note that as a substitute for compulsory vaccination, Professor Crookshank proposes compulsory notification and isolation. While the people of Leicester take particularly good care that this latter means is carried out in every detail in their own town, there is no subject on which the official organ of the Anti-Vaccination movement is fonder of fulminating than this very question of compulsory isolation, and if Professor Crookshank got his views carried into effect to-morrow, vaccination being abolished and isolation substituted, we think it very likely that the *Vaccination Inquirer* would change its name to the Isolation Inquirer, and that Anti-Vaccinationist would simply become metamorphosed into Anti-Isolationist.

